# Neural Networks Mediating High-Level Mentalizing in Patients With Right Cerebral Hemispheric Gliomas

**DOI:** 10.3389/fnbeh.2018.00033

**Published:** 2018-03-06

**Authors:** Riho Nakajima, Masashi Kinoshita, Hirokazu Okita, Tetsutaro Yahata, Mie Matsui, Mitsutoshi Nakada

**Affiliations:** ^1^Pharmaceutical and Health Sciences, Kanazawa University, Kanazawa, Japan; ^2^Department of Neurosurgery, Kanazawa University, Kanazawa, Japan; ^3^Department of Physical Medicine and Rehabilitation, Kanazawa University Hospital, Kanazawa, Japan; ^4^Institute of Liberal Arts and Science, Kanazawa University, Kanazawa, Japan

**Keywords:** mentalizing, fronto-striatal tract, superior longitudinal fascicle, awake surgery, glioma

## Abstract

Mentalizing is the ability to understand others’ mental state through external cues. It consists of two networks, namely low-level and high-level metalizing. Although it is an essential function in our daily social life, surgical resection of right cerebral hemisphere disturbs mentalizing processing with high possibility. In the past, little was known about the white matter related to high-level mentalizing, and the conservation of high-level mentalizing during surgery has not been a focus of attention. Therefore, the main purpose of this study was to examine the neural networks underlying high-level mentalizing and then, secondarily, investigate the usefulness of awake surgery in preserving the mentalizing network. A total of 20 patients with glioma localized in the right hemisphere who underwent awake surgery participated in this study. All patients were assigned to two groups: with or without intraoperative assessment of high-level mentalizing. Their high-level mentalizing abilities were assessed before surgery and 1 week and 3 months after surgery. At 3 months after surgery, only patients who received the intraoperative high-level mentalizing test showed the same score as normal healthy volunteers. The tract-based lesion symptom analysis was performed to confirm the severity of damage of associated fibers and high-level mentalizing accuracy. This analysis revealed the superior longitudinal fascicles (SLF) III and fronto-striatal tract (FST) to be associated with high-level mentalizing processing. Moreover, the voxel-based lesion symptom analysis demonstrated that resection of orbito-frontal cortex (OFC) causes persistent mentalizing dysfunction. Our study indicates that damage of the OFC and structural connectivity of the SLF and FST causes the disorder of mentalizing after surgery, and assessing high-level mentalizing during surgery may be useful to preserve these pathways.

## Introduction

Social cognition is defined as the ability to understand others’ behavior or mental state through cues such as facial expression, eye gaze, body posture and social linguistic factors (Brothers, 1990). Of these factors, the ability to understand others’ mental state is called mentalizing (Premack and Woodruff, 1978). Mentalizing processing consists of two processes, namely low-level mentalizing that works automatically, and high-level mentalizing, so called Theory of Mind that works intentionally (Apperly, 2012). Low-level mentalizing, which forms the basis of social communication, is a process that allows us to read others’ mental state through simulation of their action or facial expression (e.g., “He is angry”). High-level mentalizing is a process that allows us to predict others’ mental state from a cognitive standpoint on the basis of various cues (e.g., “He probably get offended”). This higher process involves cognitive understanding of others’ beliefs, desires, and knowledge, and understanding of others mental states. In real social life, these two subsystems always work simultaneously or mutually; they rarely work alone (Coricelli, 2005; Lieberman, 2007; Bohl and van den Bos, 2012).

Several cortical regions, such as the medial prefrontal cortex (mPFC), cingulate cortex, orbito-frontal cortex (OFC), middle frontal gyrus, temporoparietal junction, superior temporal sulcus, temporal pole, insula and amygdala, are involved in mentalizing processing, both in the right and left hemispheres, but particularly on the right side (Gallagher and Frith, 2003; Völlm et al., 2006; Carrington and Bailey, 2009; Van Overwalle, 2009; Kumfor and Piguet, 2012). In addition, different cortical areas are involved in mentalizing processing depending on types of emotion, such as automatic vs. controlled, emotional vs. neutral, positive vs. negative and affective vs. cognitive (Byrum et al., 1999; Maratos et al., 2001; Kuchinke et al., 2005; Saxe and Wexler, 2005; Keysers and Gazzola, 2007; Mukamel et al., 2010; Bohl and van den Bos, 2012; Kumfor and Piguet, 2012). Considering these facts, subcortical network of mentalizing seems to be complex and huge, but it has not been fully understood. In previous studies, some researches focused on subcortical structural connectivity mediating mentalizing. They have implicated fronto-temporo-parieto subcortical network in mentalizing processing, including the arcuate fascicle (AF), cingulum, superior longitudinal fascicle (SLF) and inferior fronto-occipital fascicle (IFOF; Philippi et al., 2009; Herbet et al., 2014; Yordanova et al., 2017). Of these white matter pathway, the AF, SLF and IFOF are known to be related to low-level mentalizing processing, while the cingulum is linked with high-level mentalizing processing (Philippi et al., 2009; Herbet et al., 2014).

Several neuroimaging studies have described the lateralization of mentalizing processes. Recent studies, including a review article, have reported the involvement of both the right and left hemispheres in mentalizing (Gallagher and Frith, 2003; Völlm et al., 2006; Carrington and Bailey, 2009; Van Overwalle, 2009; Kumfor and Piguet, 2012). Notably and in line with alternative findings, a rightward lateralization in emotional processing has been suggested by a number of studies (Schwartz et al., 1975; Borod et al., 1992, 1998; Fournier et al., 2008; Kumfor and Piguet, 2012; Yeh and Tsai, 2014; Schmitgen et al., 2016). Fusar-Poli et al. (2009) performed a meta-analysis of samples from 1600 healthy subjects and reported a rightward lateralization in emotional face processing. However, they also found that the left amygdala only was related to negative emotional processing. More interestingly, a recent study that suggested that social cognitive disorder is caused by abnormal activation of the right/left hemisphere found that a rightward lateralization pattern of brain activation was observed during social cognitive tasks in healthy individuals, while the left cerebral hemisphere was significantly activated in patients with schizophrenia and social cognitive disorder (Villarreal et al., 2014). In short, although both the right and left hemispheres are involved in mentalizing and emotional processing, the right hemisphere seems to play a more significant role than the left one.

Some recent studies have shown that both high-level and low-level mentalizing abilities decline with high possibility following glioma surgery (Herbet et al., 2013; Campanella et al., 2015; Yordanova et al., 2017). However, little is known about the postoperative recovery and long-term influence of craniotomy on mentalizing (Herbet et al., 2013). To date, emphasis has been placed on the importance of intraoperative functional preservation of emotional recognition, namely low-level mentalizing especially for right cerebral hemispheric glioma (Duffau, 2012, 2015; Yordanova et al., 2017). However, regarding higher levels of mentalizing, the necessity of preservation during surgery and possibility of postoperative recovery have not yet been demonstrated. Indeed, after the glioma surgery, sometimes disorder of higher level of mentalizing, which has large influence on patients’ social life, persist until the chronic phase (Herbet et al., 2014; Nakajima et al., 2017).

In this study, we investigated the subcortical network of high-level mentalizing in patients with right cerebral hemispheric gliomas who underwent awake surgeries. We only included patients with gliomas in the right hemisphere for the two following reasons. First, as described above, mentalizing processes seem to be lateralized on the right, and, second, preserving language function in the left hemisphere is a high priority in glioma surgery. We decided that the preservation of high-level mentalizing during awake surgery would be useful for the patients’ social lives because functional deficits in high-level mentalizing typically remain after the surgery until the chronic phase. The primary purpose of this study was to examine the neural networks underlying high-level mentalizing processing, while the secondary purpose was to investigate the usefulness of awake surgery in preserving the neural networks involved in social cognitive function, especially high-level mentalizing.

## Materials and Methods

### Patients

Patients with glioma localized in right cerebral hemisphere who underwent awake surgery in Kanazawa University Hospital between August 2013 and March 2016 participated in this study (*n* = 20; aged 47.4 ± 10.3 years; range, 31–68 years). Patient characteristics are presented in Table 1. To note, patients with recurrence at postoperative 6-month were excluded from this study. Written informed consent was obtained from all individual participants. This study was performed according to the guidelines of the Internal Review Board of the Kanazawa University, and was approved by the medical ethics committee at Kanazawa University (No.1797-2).

**Table 1:** Patients characteristics.

Case	Diagnosis	Lesion	RT, CTX	Removal	Intraop HLM test
1	Diffuse astrocytoma	Frontal	No	GTR	No
2	Diffuse astrocytoma	Frontal	No	PR	No
3	Diffuse astrocytoma	Frontal	No	GTR	No
4	Diffuse astrocytoma	Frontal	No	STR	Yes
5	Diffuse astrocytoma	Frontal	No	GTR	Yes
6	Oligodendroglioma	Frontal	No	GTR	No
7	Oligodendroglioma	Frontal	No	PR	No
8	Oligodendroglioma	Frontal	No	GTR	No
9	Anaplastic astrocytoma	Frontal	RT, CTX	GTR	Yes
10	Anaplastic astrocytoma	Frontal	RT, CTX	PR	No
11	Anaplastic astrocytoma	Frontal	CTX	GTR	Yes
12	Anaplastic oligodendroglioma	Frontal	CTX	PR	No
13	Anaplastic oligodendroglioma	Frontal	CTX	STR	Yes
14	Anaplastic oligodendroglioma	Parietal	CTX	PR	No
15	Anaplastic oligodendroglioma	Frontal	RT, CTX	GTR	Yes
16	Glioblastoma	Frontal	RT, CTX	PR	No
17	Glioblastoma	Frontal	RT, CTX	GTR	No
18	Glioblastoma	Frontal	RT, CTX	STR	No
19	Glioblastoma	Frontal	RT, CTX	GTR	Yes
20	Glioblastoma	Temporal	RT, CTX	GTR	Yes

*M, male; F, female; GTR, gross total resection; STR, subtotal resection; PR, partial removal; RT, radiotherapy; CTX, chemotherapy. To note, personal data including sex and age were excluded from the table*.

High-level mentalizing was assessed during awake surgery in eight patients who made up the “with intraoperative high-level mentalizing test (HLM test)” group. In this group, the regions with positive mapping that were elicited by direct electrical stimulation (DES) were preserved. Importantly, we resected the central part of the tumor in all patients to fulfill our oncological priorities and performed the intraoperative assessments on the extended resection using the HLM test. Twelve patients who were included in the “without intraoperative HLM test” group were not assessed with the HLM test during surgery because they underwent treatment prior to our hospital’s implementation of the intraoperative HLM test (eight cases) or they were not able to complete the test due to fatigue or other surgical events (one case). Additionally, we decided not to perform the intraoperative HLM test in three cases due to oncological reasons. No significant between-group differences, namely the with and without intraoperative HLM test group, in basic characteristics were observed: age (years), 42.1 ± 6.9; 47.9 ± 11.4; WHO grade, II 2, III 4, IV 2; II 6, III 3, IV 3; volume of resection cavity (cm^3^), 75.7 ± 58.9; 61.6 ± 28.0; total score of Mini-mental state examination (MMSE), 28.9 ± 1.5; 27.7 ± 2.5. Moreover, 18 normal healthy volunteers (aged 46.0 ± 9.0 years; total score of MMSE, 29.3 ± 1.2) were recruited as a control group, and there was no significant difference among groups.

### Neuropsychological Assessment

We used the cartoon format of the picture arrangement (PA) task of the Wechsler Adult Intelligence Scale—third edition (WAIS-III; Wechsler, 2006) as an assessment of high-level mentalizing. The WAIS-III, including the PA task, is a worldwide assessment tool for overall intelligence. The cartoon format of the WAIS PA task has been widely used in the study for social cognition (Sarfati et al., 1997; Happe et al., 1999). The task consists of 11 items, and it includes intention condition task, which is required for understanding others’ mental state or intentions, namely high-level mentalizing. During the task, 4–6 cards were presented at random in front of patients, who were asked to sort these cards into the correct order based on the story. A score was given for each item according to the WAIS-III manual (Wechsler, 2006): a score of 1 or 2 for a correct answer, and no score for incorrect answers or time-out. Through this procedure, a total score was calculated as the WAIS PA task score. All patients were assessed three times: before surgery, 1 week after surgery and 3 months after surgery. These assessments were performed by the same trained occupational therapist (R. N.). The data were collected from medical records retrospectively.

Prior to the study, we studied the relationship between the scores between the WAIS PA and the high-level mentalizing test with 65 normal healthy volunteers (aged 47.3 ± 20.8 years). The carton format HLM test we used was a typical task for high-level mentalizing, namely the false belief task (Takamiya et al., 2009). The story is as follows: “Mary, a girl, had a doll. Mary put the doll on the ground because she saw a flower and wanted to pick it. While Mary was looking at the flower, a boy (Ken) took Mary’s doll. When Mary looked for her doll where she last put it, she was surprised to find that the doll was missing”. Using Pearson’s correlation analysis, a significant positive correlation was observed (*R* = 0.65, *p* < 0.001). This result enables to confirm the adequacy of the WAIS PA task as an assessment of high-level mentalizing ability. Here are reasons why we used whole WAIS PA task to assess high-level mentalizing: (1) validity and reliability of WAIS-III including PA task has been confirmed as an entire test (Wechsler, 2006); and (2) we have found that the score of WAIS PA task well reflected high-level mentalizing accuracy from our preliminarily research as mentioned above. In addition, we performed other neuropsychological examinations including processing speed, attention, visuospatial cognition, low-level mentalizing and executive function for each patient.

### Magnetic Resonance (MR) Images

Structural MR images were acquired during the 3-month postoperative period. The images had been acquired using conventional high-resolution 3DT1-weighted sequences on a 3.0 Tesla MRI scanner (igna Excite HDx 3.0T, General Electric Medical Systems). MR images were normalized to the Montreal Neurological Institute (MNI) template using SPM12^1^ implemented in the Matlab environment^2^ and then resection cavities in all patients were reconstructed using MRIcron software^3^. Each reconstruction was first achieved by R. N. and inspected by a neurosurgeon (M. K.).

### Statistical Analysis

#### Behavioral Data

The Steel test, namely non-parametric multiple comparison analysis was used to compare time-series scores of patients (preoperative, postoperative 1 week and postoperative 3 months) with scores of healthy volunteers. Moreover, scores were compared among three groups: the with intraoperative HLM test group, without intraoperative HLM test group, and normal healthy volunteers group. To note, in this study we used non-parametric statistical analysis for patients’ data, since it did not fit well in normal probability distribution.

#### Tract-Wise Lesion Symptom Analysis

We used two kinds of tract-wise lesion symptom analysis. Recently, using white matter atlas in neuro-imaging analyses become common (Herbet et al., 2014; Thiebaut de Schotten et al., 2014), and the results obtained from them are generally consistent with the results obtained from other techniques including tractography and DES of white matter tracts during awake surgery (Thiebaut de Schotten et al., 2005).

##### Tract-based lesion-symptom (TBLS) analysis

The first step of this analysis was to estimate the amount of white matter that was resected. For this purpose, each resection cavity map was overlaid with the recent diffusion tensor imaging based white matter fiber atlas from the group of Thiebault de Schotten (Rojkova et al., 2016). Next, using MRIcron software^3^, we automatically computed the number of overlapping voxels with each associated fiber. Tract probabilities were more than the threshold of 0.5. We then performed correlation analysis by Spearman’s rho with considering Bonferroni correction to estimate the relationship between the WAIS PA score and the severity of damage of each tract due to surgical resection. Raw test score was transformed into standard residuals in which age and educational level were entered as predictors. All data were analyzed using the statistical analysis software JMP (Version 10.0.0, SAS Institute, Inc., Cary, NC, USA).

##### Disconnection analysis

To investigate the spatial location and probability of disconnection induced by DES and surgical resection, we used Tractotron software, as a part of the BCBToolkit^4^. Tracttron enables to measure the disconnected probability considering topological position of the lesion and the number of damaged voxels on specific tract. The software outputs on Excel file with a disconnected probability of each given tract. The atlas of white matter was obtained from a group of healthy controls (Rojkova et al., 2016). We performed correlation analysis with Spearman’s rho, with considering Bonferroni correction. Moreover, to visualize the spatial location of disconnected pathways, we used Disconnectome MAPS software, as a part of BCBToolkit. This test provides an estimate of the disconnection of every voxel due to the lesioned volume of interest (VOI). Disconnectome MAPS also generates a map on the NMI template and visualizations of the probabilities of white matter disconnections based on the VOIs. Here VOIs of cortical and subcortical positive mapping sites were used as the lesioned VOI. Positive mapping sites were plotted on the normalized images using medical records and intraoperative movies, making use of conventional anatomical landmarks.

#### Voxel-Based Lesion-Symptom (VLSM) Analysis

To demonstrate the putative relationship between the high-level mentalizing accuracy and the location of the resection cavity, the VLSM analysis was performed as previously described using NPM software provided in the MRIcron package (Kinoshita et al., 2016). The dependent variables were standard residuals of WAIS PA task. Only voxels that showed damage in >10% of the subjects were included (Teichmann et al., 2015; Kinoshita et al., 2016). The parametric* t*-test was chosen to generate the statistical maps. A FDR correlation was systematically applied to control false positive error, with a threshold of *p* = 0.05. The significant differences between with and without lesion were identified and presented as Z scores at MNI coordinates. To investigate the potential roles of the tracts, a standardized white matter atlas was used (Rojkova et al., 2016).

### Surgical Procedure

All patients were operated using an asleep-awake-asleep technique with cortical and subcortical brain mapping achieved by electrical stimulation (Duffau et al., 2002). After a dural incision, the cortical and subcortical areas were evaluated using DES, which was delivered via a bipolar probe (5-mm tip) with a biphasic current (pulse frequency, 60 Hz; single-pulse phase duration, 1 ms; amplitude of biphasic current, 3–6 mA). Several tasks were performed intraoperatively, and were selected carefully considering optimal onco-functional balance in each patient (e.g., picture naming and movement task, the expression recognition test namely low-level mentalizing test, the HLM test, and the line bisection test, etc.). Operative view and the intraoperative assessments were recorded on a hard disk via video camera. In the intraoperative mentalizing test, we tried to perform both low- and HLM tests to optimize the postoperative social lives of the patients. However, certain criteria, including preoperative neuropsychological examinations and intraoperative patient condition assessments, are required to perform these intraoperative neuropsychological assessments. Generally, because the HLM test is more difficult than the low-level test, the HLM test is more difficult to perform intraoperatively. Therefore, sometimes we decided to only perform the low-level mentalizing task during surgery.

### Neuropsychological Assessment During Awake Surgery

The typical task of high-level mentalizing, namely the false belief task, was used as the intraoperative HLM test as mentioned above (Takamiya et al., 2009). The method of presentation was adapted for awake surgery as described previously (Sarfati et al., 1997; see Supplementary Figure S1). First, three comic strips appeared automatically (1 strip/2 s) on PowerPoint^®^. Next, two choices were presented on the fourth strip, and patients had to choose which one of the two answers was the most logical to complete the comic strip sequence. Cortical or subcortical areas were stimulated directly when 2nd and 3rd strips are presented. Since intraoperative assessment should be uncomplicated and patients should certainly give correct response in normal condition, we decided to use the simple HLM test for intraoperative task, which is not same as pre- and post-operative assessment tool.

## Results

**Figure 1:**
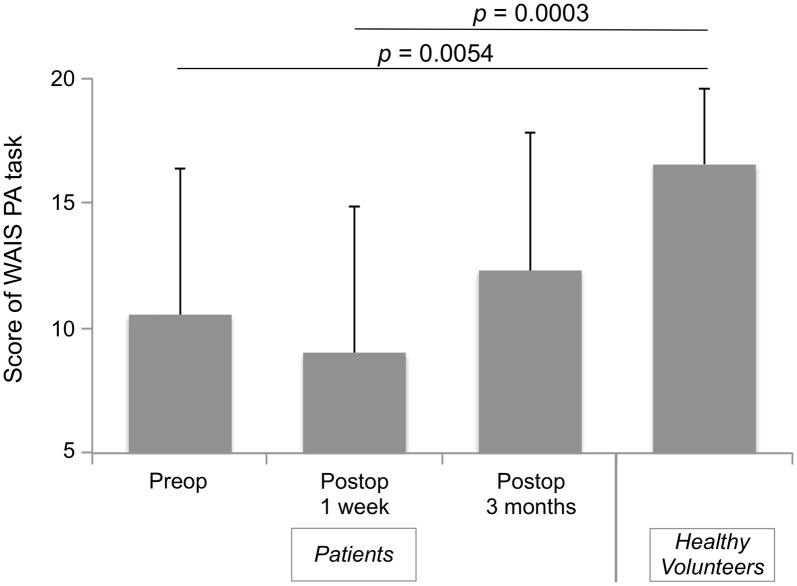
Time series of Wechsler Adult Intelligence Scale (WAIS) picture arrangement (PA) score of all patients. Bar-graph shows mean ± standard deviations of time course of patients group and healthy volunteers group. A Steel analysis (non-parametric multiple comparison analysis) was performed. Scores of all patients at preoperative and postoperative 1 week were significantly lower than those of healthy volunteers, while there was no difference between groups at postoperative 3 months.

Accuracy of high-level mentalizing in patients was lower than that of healthy volunteers before surgery and 1 week after surgery (Figure 1). However, at 3 months after surgery, there was no significant difference between the score of all patients and healthy volunteers. In the comparison of three groups, scores of both the with and without intraoperative HLM test groups were lower than that of healthy volunteers at preoperative and postoperative 1 week (Table 2). Notably, at postoperative 3 months, the normalization of the score was observed only in the with intraoperative HLM test group. Additionally, processing speed, attention, and executive function were disturbed postoperatively in some patients (4, 3 and 3 cases, respectively), but these deficits were not severe enough to disturb these patients’ performances on the WAIS PA test (Supplementary Table S1). In addition, of the six patients whose high-level mentalizing were disturbed, three showed low-level mentalizing deficit at postoperative 1 week. At the chronic phase, low-level mentalizing deficit was observed in only one patients, while high-level mentalizing deficit remained in three patients (Supplementary Table S2).

**Table 2:** Wechsler Adult Intelligence Scale (WAIS) picture arrangement (PA) score of time series of three groups.

Time	With intraop HLM test (*n* = 8)	Without intraop HLM test (*n* = 12)	Healthy volunteers
Pre-op	11.88 ± 5.24*	9.67 ± 6.43*	
Post-op 1 week	10.25 ± 5.18*	8.17 ± 6.35**	16.56 ± 3.02
Post-op 3M	13.88 ± 5.36	11.25 ± 5.72*	

A Steel analysis (non-parametric multiple comparison analysis) was performed, and mean ± standard deviation were shown in the table. Asterisks indicate that the score of With intraop HLM test group and/or Without intraop HLM test group are significantly lower than that of Healthy volunteers group; *p < 0.05, **p < 0.01.

**Figure 2:**
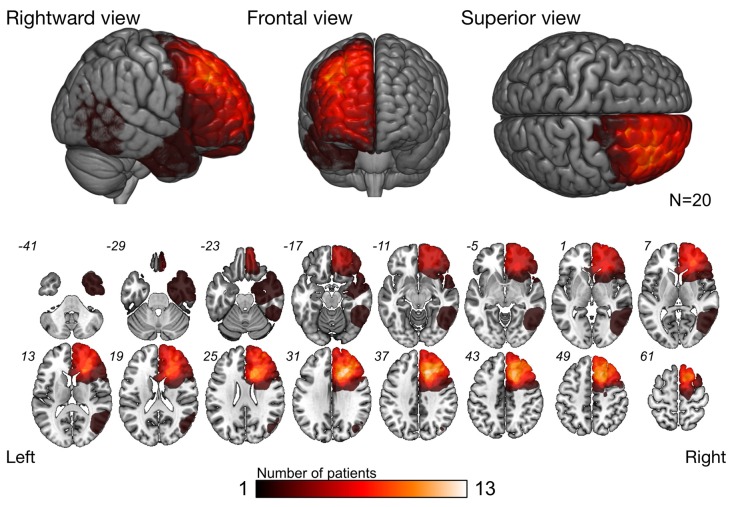
Overlap map of resected region. Overlap map of resection cavities shows that the right prefrontal cortex was the region with the greatest overlap. As for subcortical level, the greatest overlap region was equivalent to the course of the frontal-aslant tract (FAT) and fronto-striatal tract (FST).

The overlap map of resection cavity (Figure 2) demonstrates that the right medial prefrontal region was the greatest overlap (*n* = 13), and next, deep part of the cingulate cortex was the great overlap (*n* = 11). The greatest overlap of resected region was equivalent to the course of the frontal aslant tract (FAT) and fronto-striatal tract (FST).

The TBLS analysis was used to analyze the relationship between the resected lesion volumes in each association pathway (voxels) and the accuracy of the high-level mentalizing (Table 3, left row). A Spearman’s correlation analysis revealed a significant correlation between the WAIS PA scores and the cingulum anterior segment (*ρ* = −0.59, *p* = 0.0082), FST (*ρ* = −0.75, *p* = 0.0002) and SLF III (*ρ* = −0.79, *p* < 0.0001). With the Bonferroni correction, only the resected volume of the FST and SLF III remained significantly correlated with the WAIS PA scores. Importantly, we did not find any significant correlations between the overall tumor resected volumes and the postoperative WAIS PA scores at 1 week and 3 months (*ρ* = −0.29, *p* = 0.21; *ρ* = −0.16, *p* = 0.51; respectively).

**Table 3:** Correlations between social cognition accuracy and damage to white matter tracts.

White matter tracts	TBLS analysis	Disconnection analysis
AF (long segment)	−0.42	−0.41
Cingulum	−0.59**	−0.40
FAT	−0.23	−0.24
FST	−0.75***	−0.60*
IFOF	−0.49	−0.39
SLF II	−0.36	−0.20
SLF III	−0.79***	−0.56*
UF	−0.35	−0.28

Using a Spearman’s correlation analysis, correlation between social cognition accuracy and resected volume/ probability of disconnection were calculated. Each value indicate coefficient of correlation (ρ). AF, arcuate fasciculus; FAT, frontal-aslant tract; FST, fronto-striatal tract; IFOF, inferior fronto-occipital fasciculus; SLF, superior longitudinal fasciculus; UF, uncinate fasciculus. *p < 0.05; **p < 0.01; ***p < 0.001; Only results in bold survive the Bonferroni correction.

In support of these results, the disconnection analysis also indicated significant correlations between the WAIS PA scores and the FST (*ρ* = −0.57, *p* = 0.011) and SLF III (*ρ* = −0.56, *p* = 0.013; Table 3, right row). However, neither correlation remained significant after the Bonferroni correction. Anatomically, the FST and FAT run similar (but not the same) courses in the frontal lobe. Thus, it is interesting that only the FST was significantly correlated with high-level mentalizing in both analyses.

**Figure 3:**
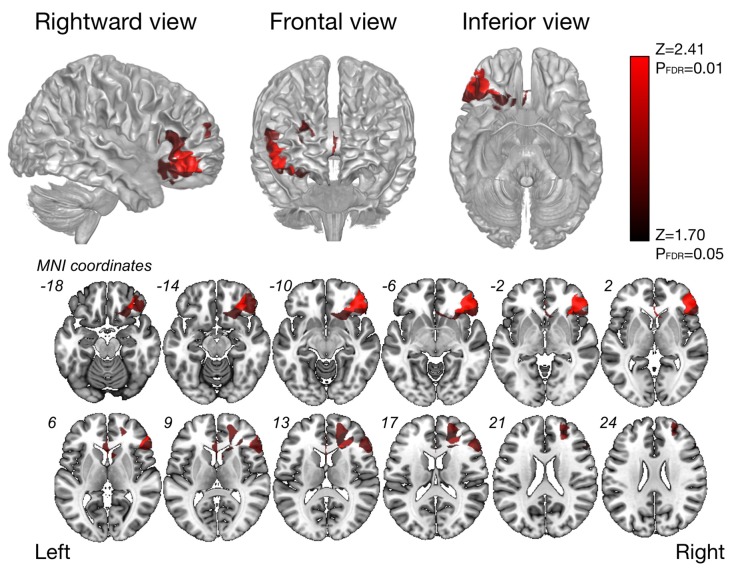
Results of the voxel-based lesion-symptom (VLSM) analysis. The VLSM analysis of high-level mentalizing accuracy was performed using resection cavity maps. Only voxels surviving on a FDR-controlled threshold (*p* = 0.05; *z* = 1.70) are shown.

The VLSM analysis for high-level mentalizing score revealed that clusters of the most significant voxels were in the inferior frontal orbital, partly in the frontal inferior triangles, which was on the course of the SLF III (cluster size, 22627; *Z*_max_ = 3.48), and second-significant voxels were in subcortical region of the middle frontal gyrus (cluster size, 6387, *Z*_max_ = 2.41; Figure 3). In our patients group, more than approximately 90% of patients include these significant voxels in resection cavity (Supplementary Table S3).

**Figure 4:**
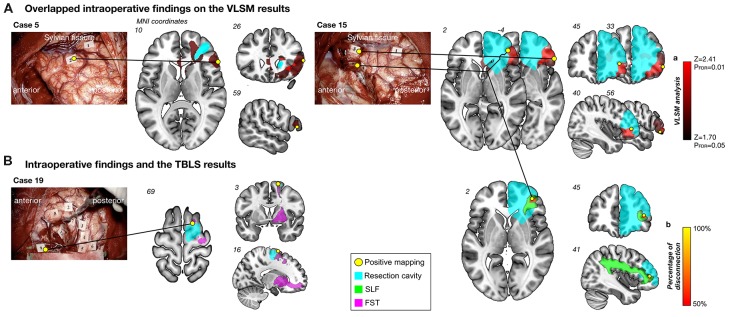
Results of intraoperative findings with the voxel-based lesion-symptom (VLSM) and tract-based lesion-symptom (TBLS) analyses. Overlapped intraoperative findings on the VLSM results **(A)**. Positive mapping sites (yellow circles) were located on the inferior frontal orbital and deep in the inferior frontal gyrus, which matched results of the VLSM analysis (red region). Numeral tags on intraoperative photographs represent results of mapping as follows; Case 15: Tag 1, motor; Tag 3 and 5, high-level mentalizing; Tag 4, visuospatial cognition, Case 5: Tag 1, dysarthria; Tag 2, high-level mentalizing. Cyan region, resection cavity. Intraoperative findings and the TBLS results **(B)**. Left column: positive mapping site (Tag 9) was found in the superior frontal gyrus, which was on the origin of the fronto-striatal tract (FST) (pink). Tag 2, 3, 4, 5 and 6, dysarthria; Tag 7 and 8, involuntary movement; Tag 9, high-level mentalizing; Tag 11, 12 and 13, positive findings using motor evoked potential. Right column: Spatial location of temporal disconnection of white matter induced by direct electrical stimulation (DES; yellow to orange region) was located along the superior longitudinal fascicles (SLF) III (green). Percentage of disconnection: yellow region, 100%; red region, 50%.

Positive mapping sites were observed in cortical and/or subcortical regions of the inferior frontal gyrus in two patients (Cases 5 and 15) during DES (Figure 4A). In addition, another positive mapping site was observed in case 19 and 15 in the superior frontal gyrus, which is the origin of the FST and SLF III, respectively (Figure 4B). Here we show an illustrative case of the intraoperative HLM test from Case 15. During stimulation to the inferior frontal orbital and deep in the inferior frontal gyrus, high-level mentalizing disorders were elicited reproducibly. Intriguingly, when these intraoperative findings were overlapped on the results of the VLSM analysis, spatial location of positive mapping sites corresponded exactly to clusters of the most significant voxels by the VLSM analysis (Figure 4A). To specifically identify the white matter disconnected temporarily induced by the DES, a disconnection map was computed for each positive mapping site. Results indicate that much of voxels with high probability to be disconnected (≥50%, above the chance level (Chechlacz et al., 2014)) were located along the SLF III (Figure 4B, right-ward column). In addition, they could have returned to their previous social life smoothly, because her high-level mentalizing ability was successfully preserved after surgery.

## Discussion

We investigated the usefulness of awake surgery for preservation of high-level mentalizing. For this, we studied the time-series of high-level mentalizing in patients with glioma localized in the right cerebral hemisphere, who were assigned to two groups: the with intraoperative HLM test group and without intraoperative HLM test group. We found that the accuracy of high-level mentalizing of the with intraoperative HLM test group was as the same as that of normal healthy controls at postoperative 3 months, while the without intraoperative HLM test group did not recover their mentalizing abilities. Moreover, our study showed that the ventral portion of the SLF and FST, and the OFC mainly played a critical role in high-level mentalizing. These findings suggest that with considering optimal onco-functional balance, implementing the intraoperative HLM test may preserve high-level mentalizing, which is essential in social life.

### Postoperative Recovery of High-Level Mentalizing

Some medical professionals have the opinion that emotional deficits recover spontaneously within a few weeks after surgery, even if cortex is damaged by surgery. In line with this clinical impression, our patient groups also recovered within 3 months following surgery, even though high-level mentalizing was disturbed before and just after surgery. In general, higher brain functions consist of multiple component, therefore they are hardly damaged completely even if one component is damaged, since other regions can compensate for damaged regions (Price and Friston, 2002). Previous neuroimaging studies have demonstrated that the medial frontal cortices, dorsal temporal cortices, basal ganglia, and amygdala are related to low-level mentalizing processing. The regions related to high-level mentalizing are more widespread and include the medial and dorsal frontal, parietal, and temporal cortices in both hemispheres, but more so on the right side (Satpute and Lieberman, 2006; Lieberman, 2007). In other words, the high-level metalizing network may more easily recover, even after the damage induced by surgery (Herbet et al., 2013). However, only patients who underwent intraoperative assessment of high-level mentalizing had the same score as healthy volunteers at 3 months. Our results might provide evidence for the usefulness of intraoperative assessment of high-level mentalizing by awake surgery. Understanding others’ mental state is a basic ability required in our social life, including empathy for others, adaptation to culture, and moral reasoning (Baimel et al., 2015). Thus, preserving high-level mentalizing by awake surgery may be important for social life of patients.

### Orbito-Frontal Cortex Plays Critical Role in High-Level Mentalizing

Results of the VLSM analysis indicated the most significant voxels were gathered largely in the OFC, and partly in the frontal inferior triangles. To note, in line with previous study (Herbet et al., 2014), we found second-significant region in deep part of the middle frontal gyrus, though statistical power was relatively low compared with the OFC. It is now well established that the OFC and mPFC including cingulate cortex regard as central component among several associated cortices for emotional and mentalizing process (Kringelbach, 2005; Philippi et al., 2009). Especially, the OFC involved in decision-making, and its damage induce disorder of social and emotional judgment of behavior (Kringelbach, 2005; Völlm et al., 2006). Moreover, together with the amygdala, the OFC plays a role in reading others emotional state from eye expression (Gallagher and Frith, 2003). However, as mentioned above, the OFC regards as the core region for mentalizing processing that works under various mental state conditions (e.g., false belief, deception, empathy and pretense) and various task condition (e.g., simple question, stories, static images, comic strips and so on; Philippi et al., 2009). Our result also provides an evidence that the OFC plays a critical role in high-level mentalizing and its damage may cause persistent mentalizing dysfunction.

### The SLF III and Cingulum Are Related to High-Level Mentalizing Processing

The SLF III is an association pathway which communicate the inferior parietal lobe and middle and inferior frontal gyri. Our data showed strong correlation between damage of the SLF III and mentalizing accuracy, and previous some direct and indirect studies support our results (Barbey et al., 2014; Herbet et al., 2014; Parkinson and Wheatley, 2014; Cabinio et al., 2015; Yordanova et al., 2017). For instance, involvement of the right SLF III to the reading the mind in the eye’s test, which one estimate other’s mental state from eye gaze of facial expression, has been reported using neuro-imaging analysis (Herbet et al., 2014; Cabinio et al., 2015). More recently, a direct evidence using DES during awake surgery revealed that along with the IFOF, the SLF/AF plays important role in accurately inferring complex mental states from human face (Yordanova et al., 2017). These previous studies indicated the involvement of the SLF III to low-level mentalizing. Recently, some studies raised the possibility that the SLF III also relates to higher level emotional process. The SLF III relates to emotional empathy, which means understanding the internal expression of others through visceral or affected reactions (Parkinson and Wheatley, 2014). In addition, anti-social behavior, namely disorder of emotional process relate to abnormality of the SLF III (Karlsgodt et al., 2015; Lee et al., 2016). Taken together with these recent reports and our results, we suggested that the SLF III plays critical role for high-level mentalizing process.

In our patient series, postoperative high-level mentalizing was disturbed in some patients, although the low-level one was normal. Patients whose low-level mentalizing was disturbed always showed high-level mentalizing deficit. This suggested not only low-level but also high-level mentalizing sites should be preserved. We speculate that certain fibers relate to both low- and high- level mentalizing, whereas there are fibers which relates to only high-level mentalizing. Since it is beyond the scope of our current study, further research is required.

In line with a previous study using the TBLS analysis (Herbet et al., 2014), our results indicated that the right cingulum anterior segment was associated with mentalizing. Although our finding was less significant, and did not survive under the Bonferroni correction, it is important to mention the involvement of the cingulum in mentalizing. Within some regions that were activated during the mentalizing tasks, the cortical regions connected by the cingulum, including the anterior cingulate cortex (ACC), mPFC, and OFC, in the right or both hemispheres are considered the “core region” of mentalizing processing (Völlm et al., 2006; Carrington and Bailey, 2009). Other functional MRI (fMRI) studies have demonstrated that the mPFC and ACC were associated with mental state, and strong activations of these regions were found during a task that required distinguishing intentions of others’ behavior (Mar, 2011). Our findings are thus consistent with the previous study focused on white matter, and functional role of their cortical terminations also support our results.

### The FST Is Related to High-Level Mentalizing Processing

The FST has been implicated in the attentional network (Chen et al., 2016), language network (Duffau et al., 2002), and network of motor control (Kinoshita et al., 2015). To best of our knowledge, this study is the first to demonstrate the involvement of the FST in mentalizing processing, whereas evidence from previous studies has indicated that cortices connected by the FST relate to networks of lower and higher levels of mentalizing (Carrington and Bailey, 2009; Schurz and Perner, 2015).

The FST plays a major role in communication between the motor area and SMA proper with the posterior putamen, and the pre-SMA with rostral parts of the striatum, through the anterior limb of the internal capsule (Lehéricy et al., 2004). In addition, the FST projects into a more extended region including the mPFC (Rojkova et al., 2016). In our patients group, the mPFC and SMA were resected with high probability within these cortices. Other neuroimaging studies have demonstrated that each cortex plays a different role in mentalizing processing. For example, activation of the SMA is observed in situations requiring prediction of others’ mental state (Carrington and Bailey, 2009; Schurz and Perner, 2015), in particular in relating to the understanding of the intentions and actions of others; so-called higher level of mentalizing (Spunt and Lieberman, 2013). As mentioned above, the mPFC is one area of a core region of mentalizing and is associated with reflective reasoning concerning actions and judgments of others (Carrington and Bailey, 2009; Schurz and Perner, 2015). Even more interestingly, the mPFC may be associated with understanding the emotional state of others (de Lange et al., 2008; Van Overwalle and Baetens, 2009), since this region is activated during mentalizing processing (Saxe and Wexler, 2005; Schlaffke et al., 2015). On the other hand, the basal ganglia seem to be involved in a lower level of mentalizing processing. The basal ganglia receive affected input from the limbic cortex which is involved in emotion and motivation (Byrum et al., 1999). Moreover, the basal ganglia automatically codes the emotional trait and evaluates implications of observed behaviors (Satpute and Lieberman, 2006; Lieberman, 2007). These previous findings regard for functional role of cortical termination of the FST support our finding that the FST is associated with mentalizing processing.

High-level mentalizing and low-level mentalizing involve a dual stream network, and interact with each other (Herbet et al., 2014). However, it is not known whether these two networks can work independently or concertedly (Bohl and van den Bos, 2012). Interestingly, our study raises the possibility that the FST, which relates to both high-level and low-level mentalizing processing, is part of a common network of mentalizing processing. Further studies are required to identify the precise role of the FST in communication between these networks.

### Study Limitation

The current study has some limitations. First, our patients group includes patients who performed radiotherapy (RT). Previous studies reported that RT cause early or late cognitive dysfunction, especially attention and executive functional deficit (Klein et al., 2002; Douw et al., 2009). Though there is no evidence that whether RT influence on emotional process, we cannot deny the possibility that RT cause early or late emotional disorder. In the same line, our patients group includes patients with glioblastoma, which is not biologically same as low and intermediate grade glioma. We have excluded the patients with recurrence at postoperative 6-month. It means tumor was well controlled at least postoperative 3-month when neuropsychological examination was performed. However, further research will be required to study differences between high and low/intermediate grade glioma regards for involvement of white matter in mentalizing. Similarly, the number of patients in the patient group was not sufficient for strong statistical power. However, some neuroimaging studies have reported successful results with a relatively small number of patients (Teichmann et al., 2015; Kinoshita et al., 2016). Additional studies with more cases are required to strengthen our current results.

As previously described, activated region in mentalizing task might be different depending on task condition in cortical level (Carrington and Bailey, 2009). Nevertheless, we used only cartoon format of the PA task to confirm high-level mentalizing accuracy, because almost all regions related to high-level mentalizing were activated during comic strip task (Carrington and Bailey, 2009). In addition, from the clinical point of view, since it is essential to take into account patients’ condition after surgery, we chose PA task as optimal postoperative assessment. However, if distinct task condition was used, results might be a little different.

The regions found to be significant in the VLSM analysis, namely, the inferior frontal gyrus and orbitofrontal cortex, mainly correspond to the location of the termination of the SLF III and partially to the uncinate fasciculus (UF). Previous studies have suggested that the UF is involved in sociality because the structural connectivity of this area is abnormal in patients with autism and antisocial disorder (Sundaram et al., 2008; Thomas et al., 2011; Sarkar et al., 2013). Moreover, emotional processes, including emotional regulation and empathy, are related to the UF (Herbet et al., 2015; Olson et al., 2015). Based on these findings, a significant correlation was expected between the resected volume of the UF and the high-level mentalizing scores in the current study. However, a significant correlation was found only between the scores and the resected volume of the SLF III. These results might have been due to the small number of patients (only seven) who had a resected UF (Supplementary Table S4). The small number of patients in this study was a methodological limitation and might explain why we did not find a significant correlation between the resected volume of the UF and mentalizing accuracy.

Finally, the high-level mentalizing of some patients was disturbed, even when intraoperative assessments were performed. Two explanations are proposed. First, the two (out of three) patients with deficits remaining until the chronic phase of our study exhibited the mentalizing disorder preoperatively (see Supplementary Table S2). Consistently, our recent study revealed that the deficits in patients with preoperative neuropsychological deficits tend to remain until the chronic phase, regardless of performance on the intraoperative assessments (Nakajima et al., 2017). Second, as mentioned above, the intraoperative HLM test was not performed on all patients to optimize oncofunctional balance and due to individual factors. Hence, we might have resected the brain region responsible for high-level mentalizing because the intraoperative assessment was not performed, in order to set the priority in the onco-functional balance on the extent of resection.

## Conclusion

The right SLF and FST appear to constitute a network of high-level mentalizing. Intraoperative assessment of high-level mentalizing may be useful tool for preserving postoperative high-level mentalizing ability.

## Author Contributions

MN and RN made conception and design of the work and drafted the article. RN, HO and MM performed neuropsychological examinations and got the data. RN and MK analyzed and interpreted the neuropsychological and imaging data. All authors critically revised the article. All authors reviewed final version of the manuscript and approved it for submission. MN supervised the study supervision.

## Conflict of Interest Statement

The authors declare that the research was conducted in the absence of any commercial or financial relationships that could be construed as a potential conflict of interest.

## Footnotes

^1^http://www.fil.ion.ucl.ac.uk/spm/software/spm12/

^2^http://www.mathworks.com/products/matlab/

^3^http://www.mccauslandcenter.sc.edu/mricro/mricron/

^4^http://www.brainconnectivitybehaviour.eu

## Supplementary Material

FIGURE S1 Schema of the intraoperative HLM test.
