# Supplementary material for: Neural Networks Mediating High-Level Mentalizing in Patients With Right Cerebral Hemispheric Gliomas
Source: Front Behav Neurosci. 2018 Mar 6;12:33. doi: 10.3389/fnbeh.2018.00033 (PMC5845682; doi:10.3389/fnbeh.2018.00033)
Supplement: Supplementary file 1 [file Table_1.DOCX]

**Supplementary table 1. Score of executive function**

| **Case No.** | **LCT**  **(sec)** | **LCT**  **(%correct)** | **Stroop test part1**  **(sec)** | **Stroop test part3**  **(sec)** | **Stroop test**  **Part3-1 (sec)** | **TMT-A**  **(sec)** | **TMT-B**  **(sec)** | **TMTB-A**  **(sec)** | **TMTA/B**  **(ratio)** | **Functional disorder** |
| --- | --- | --- | --- | --- | --- | --- | --- | --- | --- | --- |
| 1 | 144 | 100.0 | 17 | 25 | 8 | 147 | 151 | 4 | 1.03 | PS |
| 2 | 136 | 87.7 | 19 | 36 | 17 | 149 | 198 | 49 | 1.33 | PS |
| 3 | 100 | 77.2 | 16 | 21 | 5 | 226 | 458 | 232 | 2.03 | attention |
| 4 | 98 | 99.1 | 13 | 16 | 3 | 90 | 145 | 55 | 1.61 |  |
| 5 | 134 | 91.2 | 20 | 27 | 7 | 153 | 205 | 52 | 1.34 |  |
| 6 | 93 | 96.5 | 18 | 16 | -2 | 90 | 65 | -25 | 0.72 |  |
| 7 | 103 | 90.4 | 14 | 15 | 1 | 144 | 97 | -47 | 0.67 |  |
| 8 | 106 | 56.1 | 20 | 47 | 27 | Unable | Unable | NA | NA | attention |
| 9 | 94 | 97.4 | 16 | 18 | 2 | 55 | 109 | 54 | 1.98 |  |
| 10 | 155 | 95.6 | 28 | 42 | 14 | 228 | 682 | 454 | 2.99 | PS, Executive function |
| 11 | 125 | 100.0 | 22 | 16 | -6 | 142 | 195 | 53 | 1.37 |  |
| 12 | 160 | 62.3 | 23 | 26 | 3 | 245 | 347 | 102 | 1.42 | attention |
| 13 | 109 | 98.2 | 14 | 19 | 5 | 122 | 236 | 114 | 1.93 | Executive function |
| 14 | 141 | 97.4 | 15 | 18 | 3 | 140 | 154 | 14 | 1.10 |  |
| 15 | 90 | 100.0 | 14 | 17 | 3 | 118 | 94 | -24 | 0.80 |  |
| 16 | 162 | 100.0 | 12 | 17 | 5 | 110 | 120 | 10 | 1.09 |  |
| 17 | 234 | 91.2 | 27 | 31 | 4 | 314 | Unable | NA | NA | PS, executive function |
| 18 | 83 | 88.6 | 13 | 15 | 2 | 124 | 115 | -9 | 0.93 |  |
| 19 | 127 | 98.2 | 18 | 30 | 12 | 164 | 209 | 45 | 1.27 |  |
| 20 | 109 | 99.1 | 17 | 21 | 4 | 90 | 131 | 41 | 1.46 |  |
| **Age related healthy Japanese** | | |  |  |  |  |  |  |  |  |
| Mean ± SD | 85.1 ± 25.4 | 92.5 ± 20.0 | 14.7 ± 1.2 | 22.3 ± 6.8 | 7.3 ± 6.5 | 87.2 ± 27.9 | 121.2 ± 48.6 | 34 | 1.38 |  |

LCT, letter cancellation test; TMT, trail making test Japanese version; PS, processing speed; SD, standard deviation; Unable, unable to complete certain test

**Supplementary table 2. Row score of high- and low-level mentalizing**

|  | **High-level mentalizing** | | | | | **Low-level mentalizing** | | | | | |
| --- | --- | --- | --- | --- | --- | --- | --- | --- | --- | --- | --- |
|  | **Pre-op** | **Post-op**  **1 week** | | **Post-op**  **3 months** | | **Pre-op** | | **Post-op**  **1 week** | | **Post-op**  **3 months** | |
| 1 | 18 | 10 | | 14 | | NA | | 22 | | 23 | |
| 2 | 6 | ***4*** | | 10 | | ***11*** | | 17 | | 17 | |
| 3 | 16 | 12 | | 14 | | 21 | | 20 | | 20 | |
| 4 | 18 | 14 | | 20 | | 22 | | 27 | | 27 | |
| 5 | 11 | 12 | | 13 | | 24 | | 22 | | 25 | |
| 6 | 18 | 12 | | 19 | | 15 | | 27 | | 28 | |
| 7 | 20 | 22 | | 16 | | 20 | | 19 | | 20 | |
| 8 | ***4*** | ***0*** | | 6 | | 18 | | ***9*** | | 17 | |
| 9 | 16 | 8 | | 18 | | 21 | | 17 | | 22 | |
| 10 | ***4*** | ***2*** | | ***5*** | | ***11*** | | ***9*** | | ***7*** | |
| 11 | 16 | 8 | | 16 | | 18 | | 22 | | 24 | |
| 12 | ***2*** | ***2*** | | ***4*** | | ***12*** | | ***14*** | | 18 | |
| 13 | ***2*** | ***2*** | | 6 | | 21 | | 20 | | 21 | |
| 14 | 8 | 10 | | 14 | | 19 | | 19 | | 21 | |
| 15 | 10 | 18 | | 18 | | 22 | | 20 | | 19 | |
| 16 | 14 | 10 | | 18 | | 20 | | 24 | | 20 | |
| 17 | 6 | ***2*** | | ***2*** | | 17 | | ***12*** | | 21 | |
| 18 | 8 | 12 | | 13 | | 20 | | 20 | | 20 | |
| 19 | 8 | 6 | | 6 | | 16 | | 16 | | 16 | |
| 20 | 6 | 14 | | 14 | | 20 | | 20 | | 23 | |
| **Age related healthy Japanese** | | |  | |  | |  | |  | |  |
| Mean ± SD | 15.8 ± 3.8 |  | |  | | 20.1 ± 2.5 | |  | |  | |

Following tests were performed for assessments: The Expression recognition test for low-level mentalizing, and the picture arrangement (PA) test of WAIS-III for high-level mentalizing. NA, not available; ***Bold italic***, impaired (Cut-off score: WAIS PA score ≤ 5; Expression recognition score ≤ 15)

**Supplementary table 3.** Percentage of patients with at least 1, 100 or 500 voxels resected (all patients)

| **Resected voxels/ *aal*’s lavel** |  | **Frontal middle** | **Frontal middle orbital** | **Frontal inferior orbital** | **Frontal inferior triangles** | **Including**  **at least**  **one region** |
| --- | --- | --- | --- | --- | --- | --- |
| At least 1 |  | 89.5 | 26.3 | 36.8 | 79.0 | 94.7 |
| At least 100 |  | 89.5 | 26.3 | 31.6 | 68.4 | 94.7 |
| At least 500 |  | 89.5 | 26.3 | 26.3 | 63.2 | 94.7 |

Inferior significant clusters from the VLSM analysis include include frontal middle and inferior orbital cortex and frontal inferior triangles. The right row indicates the percentage of patients whose damaged regions include at least one out of three within inferior significant clusters

**Supplementary table 4. Resected volume (voxels) of all patients**

| **Case No.** | **AF (long)** | **Cingulum** | **FAT** | **FST** | **IFOF** | **SLF II** | **SLF III** | **UF** |
| --- | --- | --- | --- | --- | --- | --- | --- | --- |
| 1 | 197 | 15931 | 28246 | 30048 | 1515 | 25951 | 5458 | 0 |
| 2 | 0 | 1443 | 19913 | 11371 | 0 | 18900 | 2415 | 0 |
| 3 | 1302 | 7570 | 28783 | 23544 | 229 | 30541 | 11548 | 0 |
| 4 | 0 | 6081 | 0 | 9334 | 2217 | 121 | 1421 | 0 |
| 5 | 0 | 3 | 0 | 4261 | 3414 | 674 | 3205 | 509 |
| 6 | 291 | 607 | 5387 | 5053 | 26 | 9032 | 6203 | 0 |
| 7 | 0 | 6437 | 14114 | 14799 | 0 | 4715 | 0 | 0 |
| 8 | 7 | 5978 | 21135 | 14335 | 0 | 14797 | 123 | 0 |
| 9 | 17 | 10624 | 0 | 38721 | 22751 | 1094 | 9995 | 10882 |
| 10 | 0 | 7701 | 13366 | 13521 | 0 | 8080 | 0 | 0 |
| 11 | 315 | 18989 | 14976 | 63162 | 32310 | 13292 | 32355 | 10762 |
| 12 | 115 | 5770 | 20154 | 14802 | 0 | 21982 | 3251 | 0 |
| 13 | 18 | 6547 | 1365 | 33677 | 26658 | 3667 | 21619 | 4586 |
| 14 | 5828 | 0 | 0 | 0 | 5080 | 3683 | 0 | 0 |
| 15 | 384 | 22636 | 19890 | 60895 | 20691 | 12785 | 18039 | 8772 |
| 16 | 250 | 11569 | 24695 | 24158 | 1031 | 18045 | 7362 | 0 |
| 17 | 23 | 20309 | 9412 | 50619 | 22824 | 6499 | 17131 | 5014 |
| 18 | 1815 | 2911 | 18771 | 14645 | 79 | 24091 | 13260 | 0 |
| 19 | 0 | 0 | 1400 | 3452 | 0 | 1010 | 0 | 0 |
| 20 | 0 | 0 | 78 | 0 | 1019 | 0 | 0 | 8019 |

AF (long), long segment of the arcuate fascicles; FAT, frontal aslant tract; FST, fronto-striatal tract; IFOF, inferior fronto-occipital fascicle; SLF, superior longitudinal fascicle; UF, uncinate fascicle
