# Supplementary figures and images for: Neural Networks Mediating High-Level Mentalizing in Patients With Right Cerebral Hemispheric Gliomas
Source: Front Behav Neurosci. 2018 Mar 6;12:33. doi: 10.3389/fnbeh.2018.00033 (PMC5845682; doi:10.3389/fnbeh.2018.00033)

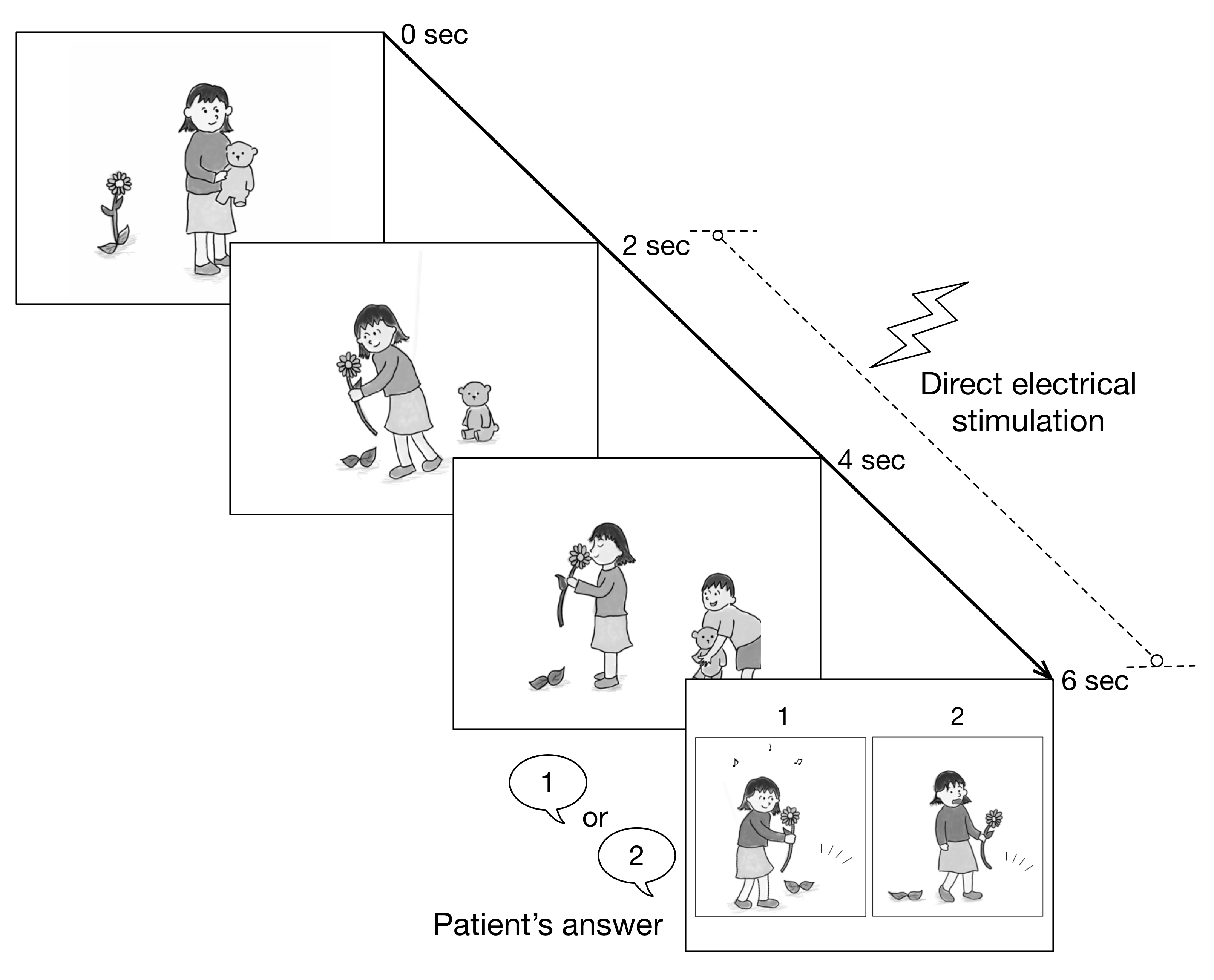

Supplement: FIGURE S1 — Schema of the intraoperative HLM test. [file Image_1.TIFF]
